# Effect of maintaining apical patency with a CM instrument on apical transportation and centering ability

**DOI:** 10.1590/1807-3107bor-2025.vol39.112

**Published:** 2025-11-07

**Authors:** Caroline Carvalho dos SANTOS, Stephanie Isabel DÍAZ ZAMALLOA, Giulio GAVINI, Israel CHILVARQUER, Celso Luiz CALDEIRA

**Affiliations:** (a) Universidade de São Paulo - USP, School of Dentistrry, Department of Dentistry, São Paulo, SP, Brazil.; (b) Universidade de São Paulo - USP, School of Dentistrry, Department of Stomatology, São Paulo, SP, Brazil.

**Keywords:** Endodontics, Cone-Beam Computed Tomography, Dental Pulp Cavity

## Abstract

The aim of this study was to assess the impact of the apical patency technique on apical transportation and centering ability of a controlled memory (CM) instrument in the apical region using cone-beam computed tomography (CBCT). Sixty distobuccal canals of extracted maxillary molars were assigned to three groups (n = 20) based on the patency length achieved using the Easy ProDesign Logic (EPL) 25.01 file: Group A — 1 mm beyond the apical foramen; Group B — at the apical foramen; and Group C — 1 mm short of the apical foramen (no patency). Each group was then subdivided into two subgroups (n = 10) according to the working length used for root canal preparation with the EPL 25.05 file: A1, B1, C1-I, and C1-II — 1 mm short of the apical foramen and A2, B2 — at the apical foramen. CBCT images were acquired at three time points: pre-patency, post-patency, and post-instrumentation. The scanned images were analyzed using the E-VOL DX software. No statistically significant difference in apical transportation was found between the groups after patency or after instrumentation (p < 0.05), irrespective of the measurement levels (0.5, 1, and 2 mm short of the apical foramen). A significant difference in the centering ability of the patency instrument was observed only at 2 mm short of the foramen (p < 0.05). The B2 group exhibited a higher centering ability, with a statistically significant difference compared to the A2 group (p < 0.05), observed only at 0.5 mm short of the foramen. In conclusion, maintenance of apical patency using the EPL instrument had no influence on apical transportation; however, it may slightly affect the centering ability of the root canal.

## Introduction

Several techniques have been employed in endodontics, and apical patency remains a topic of ongoing debate among authors concerning its advantages and disadvantages.^1-5^

Patency involves maintaining the apical portion of the canal free from debris by recapitulating the apical foramen with a small-diameter instrument.^1^ In the original technique, a small-diameter K file (#08 or #10), with a small metallic mass, is passively advanced from 0.5 to 1 mm across the apical constriction without inducing apical enlargement.^6,7^

Additionally, the apical foramen deviates from the apical center, emerging laterally to the apex,^2^ which can cause the patency instrument to work more frequently on one dentin wall than on others in the apical region, especially in curved root canals. These circumstances may lead to apical transportation and potential damage to the foramen.^8^ Consequently, apical transportation can also adversely affect the cleaning and filling of the apical portion of the root canal, compromising infection control .^9^

When not properly performed, patency procedures may result in the extrusion of root canal contents, contribute to inadequate apical sealing, cause postoperative pain, and negatively affect periapical tissues.^10^

Currently, heat-treated nickel-titanium instruments, such as controlled memory (CM) files, are preferred for apical patency procedures because of their higher flexibility and improved fatigue resistance, preserving the original root canal morphology.

Cone-beam computed tomography (CBCT) has become a reliable method for evaluating the apical transportation and centering ability of the instrument in the root canal. In addition to remarkable technological advances in tomographic software programs,^3^ CBCT now offers enhanced precision, making it one of the best non-invasive tools for quantitative and qualitative three-dimensional assessments, providing sequential axial images and detailed visualization of the internal root canal anatomy.^4^ The implementation of CBCT in endodontics also brought the noteworthy advantage of enabling clearer characterization of various research designs and approaches.^11,12^

While heat-treated instruments may minimize the controversial negative consequences of the apical patency technique, further investigation into the use of CBCT is warranted to determine the extent of apical transportation and its influence on the behavior of the instrument utilized for subsequent canal preparation. Therefore, the objective of the present study was to evaluate the impact of apical patency procedures and subsequent canal preparation with a CM instrument on apical transportation and centering ability.

## Methods

This study was approved by the Research Ethics Committee of the School of Dentistry of University of São Paulo, Brazil, under the protocol number CAAE: 95922718.0.0000.0075.

Sixty human maxillary molars with anatomically independent distobuccal canals were selected for this study. The teeth were assessed clinically and radiographically to ensure adherence to the inclusion and exclusion criteria. Only maxillary molars (excluding third molars) with fully developed apices and root curvature up to 40º were included. Teeth presenting previous endodontic treatment, pulp calcification, root fractures, or root resorptions were excluded.

The angle of the root canal curvature was measured according to the Schneider method, using a digital image processing program (ImageJ - National Institutes of Health, Maryland, USA).^13^

The teeth were cleaned with ultrasonic tips (Jet Sonic Bp - Gnatus, Ribeirão Preto, Brazil) and kept in saline solution (LBS Laborasa, São Paulo, Brazil) until the beginning of the experiment.

### Preparation of teeth and group allocation

All root canal were prepared by a single operator (CCS), an experienced endodontist previously trained to work with the instruments used in this study. Access cavities were prepared with 1014 HL spherical diamond burs (KG Sorensen, São Paulo, Brazil) and Endo-Z drills (Maillefer, Ballaigues, Switzerland) mounted on a high-speed handpiece with air and water spray cooling.

Initial exploration was performed with a # 08 K file (Dentsply Maillefer, Ballaigues, Switzerland) until the file tip was visible at the major apical foramen (located approximately 0.5 mm short of the anatomical apex) under a surgical microscope at 8x magnification The apical limit for patency and instrumentation was then determined.

**Figure 1:**
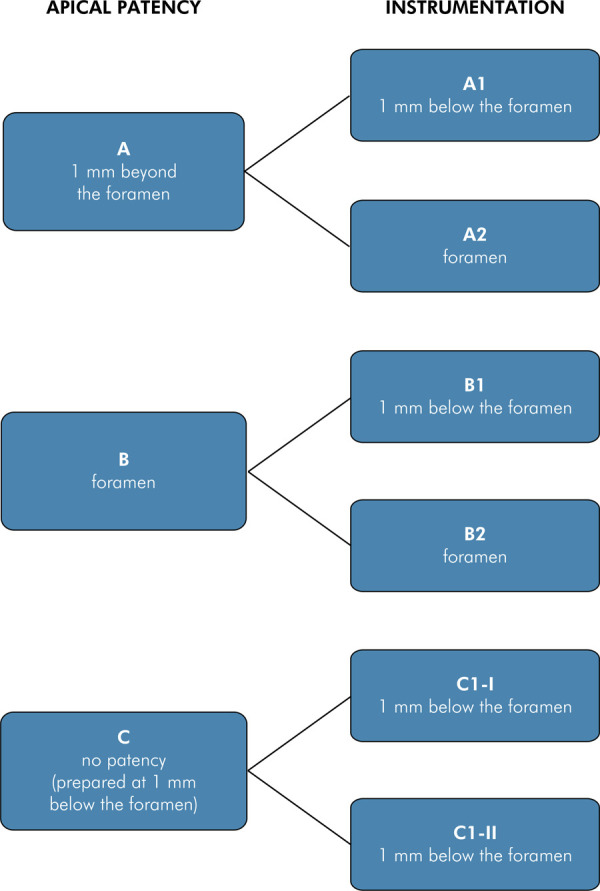
Distribution of specimens into groups according to the working length set for the apical patency instrument and to the different working lenghts for mechanical preparation. Group A1: working length of the apical patency instrument 1 mm beyond the foramen and of the mechanical preparation instrument 1 mm short of the foramen; Group A2: working length of the apical patency instrument 1 mm beyond the foramen and of the mechanical preparation instrument at the foramen; Group B1: working length of the apical patency instrument at the foramen and of the mechanical preparation instrument 1 mm short of the foramen; Group B2: working length of the apical patency instrument and mechanical preparation instrument at the foramen; Group C1-I and Group C1-II: apical patency was not performed; the working length of the patency and mechanical preparation instruments was set at 1 mm short of the foramen.

The 60 specimens were randomly assigned to three groups (n= 20) (Figure 1) based on the working length designated for the apical patency instrument.

### Image acquisition

All specimens were subjected to a pre-patency CBCT scan. CBCT images were obtained using the PreXion 3D (PreXion Inc., Yoshida Dental, Japan) with the following settings: voxel size of 0.1 mm; field of view of 5 cm; Hi-res/Hi-density; tube voltage of 90 kVp; tube current of 4 mA; and exposure time, of 37 ss. The teeth were mounted on a specific support to ensure identical position during scanning.

### Apical patency

Root canals were filled with 1% sodium hypochlorite (Farmácia Fórmula e Ação, São Paulo, Brazil) and Easy ProDesign Logic (EPL) 25/.01 instruments (BassiEndo, Belo Horizonte, Brazil) were activated using the VDW Silver® electric motor (VDW, Munich, Germany) (350 rpm and 1 N), with gentle back-and-forth movements until the instruments reached the patency length established for each group. A new instrument was used for every two specimens, and irrigation was performed with a total volume of 5 mL of 1% sodium hypochlorite. Subsequently, root canals were dried with sterile paper points and subjected to post-patency CBCT scanning, using the same parameters as those of the initial scan.

### Mechanical preparation

Six subgroups were established (n = 10) (Figure 1) for mechanical preparation, according to the working lengths for preparation. The specimens were first assigned to three groups based on the working length of the apical patency instrument: Group A — 1 mm beyond the foramen; Groups B and C — at the foramen. These groups were subdivided for canal instrumentation: 1 mm short of the foramen (1) and at the foramen (along the total length of the root canal) (2).

Note that in group C (n = 20), patency was performed 1 mm short of the foramen, and the same working length was maintained. Exceeding the working length would not simulate the actual clinical scenario; therefore, the specimens were split into subgroups I and II to facilitate the statistical analysis.

The roots were coated with condensation silicone (Profile - Coltene, Altstätten, Switzerland) to retain the intracanal irrigant, thus preventing leakage through the apical foramen. Initially, the root canal was flooded with sodium hypochlorite, followed by preparation using the EPL system 25/.05 operated with a VDW Silver® motor (at 950 rpm and 4 N). Gentle 1 mm gentle back-and- forth movements were applied in the apical direction, according to the manufacturer’s instructions, until the working length established for each group was reached. A new instrument was used for every two specimens, and irrigation was performed with a total volume of 5 mL of 1% sodium hypochlorite.

Thereafter, the root canal was irrigated with 5 mL of 1% sodium hypochlorite, followed by 5 mL of 17% EDTA-T (Farmácia Fórmula e Ação, São Paulo, SP) and 5 mL of 1% sodium hypochlorite. The root canals were dried with sterile paper points, and the condensation silicone was removed to allow for the third tomographic scan (post-instrumentation CBCT).

### CBCT image analysis

The images were reconstructed into a three-dimensional dataset and exported in DICOM format (Digital Imaging and Communications in Medicine) for analysis and optimization using the E-VOL DX software (CDT Software, São Paulo, Brazil). The specimens were mounted on a utility wax support (Wilson Polidental, São Paulo, SP), following a standardized order for repositioning during each scan.

The tomographic images were assessed and measured by the same operator (CCS), who prepared the root canals and had been previously trained in the use of the software.

### Analysis of results

Apical transportation and centering ability of the instrument were measured before patency, after patency, and after instrumentation at different root canal levels (0.5 mm, 1 mm, and 2 mm short of the apical foramen). The images were evaluated in three different planes (axial, coronal, and sagittal), and synchronization was required for comparison between the first (reference) and the second (target) images. The initial images (pre-patency) were compared with the post-patency images and, subsequently, post-patency images were compared with post-instrumentation images. Images of each specimen were analyzed, and linear measurements were performed using the E-VOL DX image processing software. The images were aligned with respect to the center of the root, maintaining the exact same position in both the pre-analysis and post-analysis. Furthermore, visualization of the images was optimized utilizing zoom tools and by adjustments of brightness and contrast parameters.

**Figure 2:**
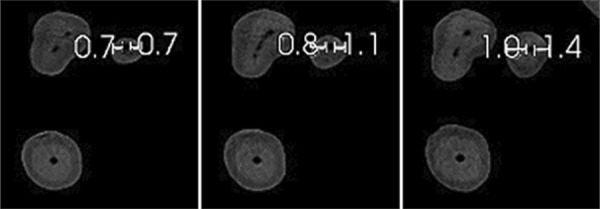
Image simulating the measurements taken in the axial sections for apical transportation and centering ability in specimen 5 of Group A (post-patency) at 0.5 mm, 1 mm, and 2 mm (from left to right).

Apical transportation and centering ability were calculated using the technique developed by Gambill et al.^4^based on axial slice measurements (Figure 2) at 0.5 mm, 1 mm, and 2 mm short of the apical foramen. Apical transportation (in mm) corresponded to a post-patency or post-instrumentation deviation of the canal from its natural axis.

**Figure 3:**
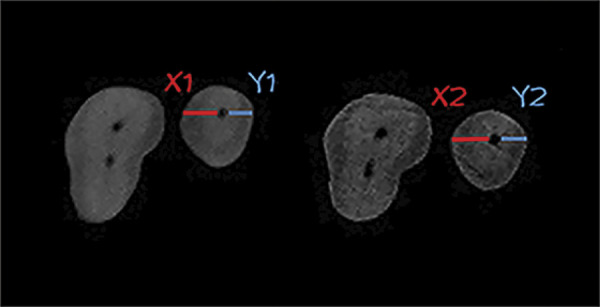
Representative image illustrating the Gambill formula: (X1 - X2) - (Y1 - Y2), where X1 represents the shortest distance from the mesial edge of the non-instrumented root canal to the mesial edge of the root, Y1 is the shortest distance from the distal edge of the non-instrumented root canal to the distal edge of the root, X2 is the shortest distance from the mesial edge of the instrumented root canal to the mesial edge of the root, and Y2 is the shortest distance from the distal edge of the instrumented root canal to the distal edge of the root.

The measurements of the reference and target images were compared to assess the magnitude and direction of apical transportation using the following formula: (X1 - X2) - (Y1 - Y2) (Figure 3), where X1 represents the shortest distance from the mesial edge of the non-instrumented root canal to the mesial edge of the root; Y1 is the shortest distance from the distal edge of the non-instrumented root canal to the distal edge of the root; X2 is the shortest distance from the mesial edge of the instrumented root canal to the mesial edge of the root; and Y2 is the shortest distance from the distal edge of the instrumented root canal to the distal edge of the root. The direction of transportation was evaluated based on the values obtained for each specimen. A negative value indicated transportation to the distal portion of the root, while a positive value indicated transportation to the mesial portion of the root. A zero value indicated absence of root canal transportation.

The mean centering ratio indicated the instrument’s ability to maintain its position along the central axis of the root canal. Each section was calculated using the formula (X1 - X2)/(Y1 - Y2), with the order reversed when necessary to ensure that the numerator represented the smaller of the two differences.

A value of 1 indicated perfect centering, whereas values closer to zero stood for worse centering ability.

Statistical differences in the measurements were assessed using the Kruskal-Wallis test, with the significance level set at 5%.

## Results

Comparisons were made between pre-patency and post-patency images and between pre-patency and post-instrumentation images, at different levels, to evaluate apical transportation and centering ability. The medians and standard deviations of apical transportation and centering ability measurements, expressed in millimeters, were tabulated separately at 0.5 mm, 1 mm, and 2 mm short of the foramen.

### Apical transportation

**Figure 4:**
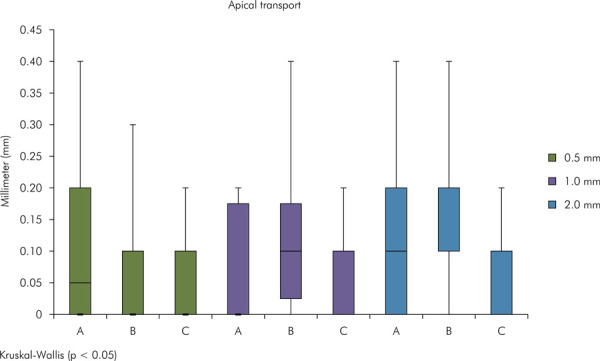
Graph illustrating apical transportation after apical patency. Different uppercase letters indicate significant difference between the experimental groups (Kruskal-Wallis test, p < 0.05).

**Figure 5:**
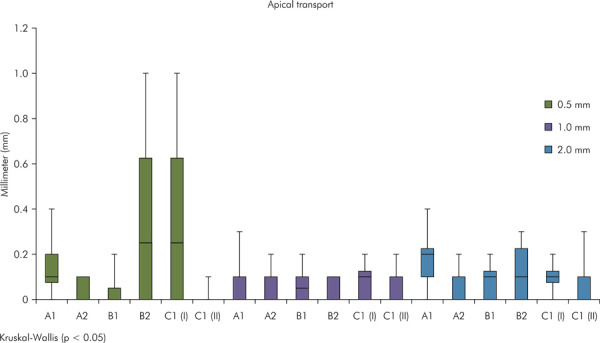
Graph illustrating apical transportation after mechanical preparation. Different uppercase letters indicate significant difference between the experimental groups (Kruskal-Wallis test, p < 0.05).

No statistically significant difference (p < 0.05) in apical transportation was observed after patency and after instrumentation at all levels (Figures 4 and 5).

### Centering ability

**Figure 6:**
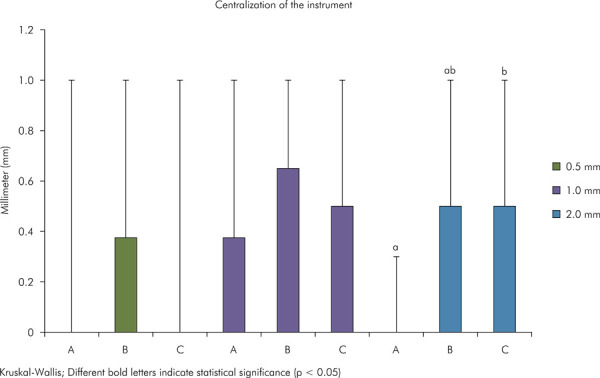
Graph representing the centering ability of the instrument after apical patency. Different uppercase letters indicate significant difference between the experimental groups (Kruskal-Wallis test, p < 0.05).

**Figure 7:**
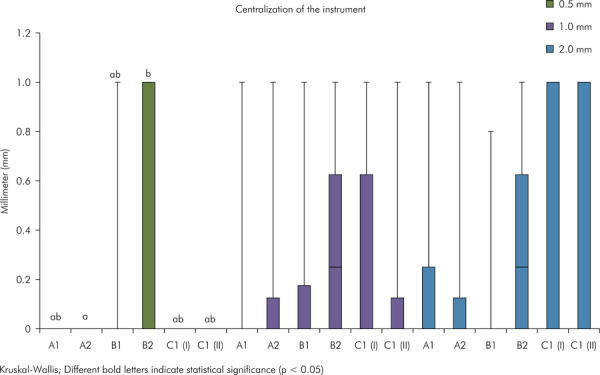
Graph representing the centering ability of the instrument after mechanical preparation. Different uppercase letters indicate significant difference between the experimental groups (Kruskal-Wallis test, p < 0.05).

After patency, A statistically significant difference (p < 0.05) in centering ability was found after patency between groups A and C at 2 mm (Figure 6). A statistically significant difference (p < 0.05) was observed after instrumentation between groups A2 and B2 at 0.5 mm (Figure 7).

## Discussion

The null hypothesis of the present study was accepted, given that no significant difference was observed in apical transportation after apical patency and instrumentation. On the other hand, the null hypothesis raised for centering was rejected, as apical patency may cause slight instrument deviation within the root canal, likely influencing the path of subsequent instrumentation.

CBCT was chosen as the method for root canal assessment, considering that no previous studies have evaluated the effects of apical patency using CBCT. CBCT is a valuable tool for examining tooth anatomy, offering high-resolution three-dimensional images both preoperatively and postoperatively, thus allowing for the assessment of transportation and centering ability after root canal preparation in the chosen cross section.^3,14^ In addition to its advantages, such as low radiation dose with a limited field of view, thus allowing for delimitation of the region of interest. This ability to limit the field of view helps enhance image resolution, enabling accurate visualization of the structure of interest.^15^

A similar study on root canal transportation using CBCT with different endodontic systems assessed multiple apical sections , achieving its objective, despite the use of a 0.2 mm slice thickness.^16^ The Prexion 3D tomograph was used in the present study to capture the images because it provides images with a voxel size of 0.1 mm. Gambill et al.^4^and Moazzami et al.^15^reported that lower the voxel sizes yield higher image resolution, allowing for precise detection of subtle changes in the root canal system.

Transportation and centering ability were assessed as linear parameters through axial sections generated with the E-VOL DX software.^3,4,14,15,17,18^ The tomographic software allows for reorientation of the scan based on the inclination of each tooth and enhances image resolution through adjustable parameters, such as brightness and contrast, sharpness, slice thickness, and volume. It also offers image filters and an advanced noise reduction algorithm. The use of submillimeter voxel sizes contributes to high-resolution images. Additionally, the software is compatible with all contemporary CBCT scanners and allows for the export of DICOM files.^11,12^

In the present study, contrast, brightness, and density were adjusted in each group to enhance the visualization of root canal contours. In addition to density and contrast adjustments, the E-VOL DX tomographic software offers control over the multi-CDT, sharpen, and radius functions, which reduce image noise and improve image accuracy.^11,12^

The E-VOL DX software features a tool that obtains actual-size measurements of anatomical structures, with an accuracy up to 0.001 mm. Consequently, the available image adjustment tools enhance the visualization of the margins of the observed, yielding precise measurements and ensuring faithful representation.^11,12^

The effectiveness of endodontic instruments in maintaining the original shape of the anatomical canal and the position of the apical foramen with minimal enlargement was used as a parameter. This concern led to the investigation of the instruments recommended for apical patency, a procedure that should preferably be performed with a flexible and small-diameter instrument.^6^

The limited number of studies evaluating new endodontic instruments for apical patency provided the rationale for the present investigation. Consequently, the EPL system was chosen based on the manufacturer’s recommendation, including an initial patency instrument (25.01), followed by an instrument with the same tip diameter but with a larger taper (25.05) for instrumentation.

The mechanical properties and behavior of this instrument have been investigated by some authors. Pinheiro et al.^19^ evaluated apical and cervical transportation after instrumentation with the ProTaper Gold, ProDesign S, HyFlex CM, HyFlex EDM, and EPL systems. While the difference was not statistically significant, the EPL system exhibited the lowest apical transportation. The findings of the present study corroborate those of previous research, with no statistically significant difference in apical transportation at different root canal levels. These findings reinforce the effectiveness of heat-treated instruments in producing canal preparations with minimal transportation, consistent with the findings of other studies.^20,21,22,23^ The flexibility imparted by the CM-Wire alloy is likely one of the factors contributing to low transportation.^18^

In the present study, three different apical patency lengths and two instrumentation lengths were selected, considering the lack of consensus in the literature regarding the working limit within the root canal.

As for the centering ability of the instrument, the present study demonstrated a significant difference (p < 0.05) at 2 mm short of the apical foramen between groups A and C. This finding indicates that maintaining apical patency 1 mm beyond the foramen may promote greater deviation of 25.01 instrument compared to performing patency 1 mm short of the foramen. This deviation may have occurred because of the anatomy of the distobuccal canal at 2 mm from the foramen, where the diameter ranges from 0.25 to 0.33 mm.^5^ At 2 mm short of the apical foramen, The EPL 25.01 instrument in group A exhibited a diameter of 0.28 mm, whereas the corresponding diameter in group C was 0.26 mm. This difference in diameter could explain the reduced centering ability observed in group A.

A significant difference (p < 0.05) in centering ability was found after instrumentation at 0.5 mm short of the apical foramen between groups B2 and A2. The root canal anatomy at this level may explain these findings because the EPL 25.05 instrument in group B2 would have a diameter of 0.275 mm at 0.5 mm, while the 25.01 instrument touched the same region, reaching a diameter of up to 0.255 mm after patency. In group A2, the patency instrument extended over 1 mm beyond the diameter at the level reached by the instrument, contacting an area with an estimated diameter of 0.265 mm. Following instrumentation at 0.5 mm short of the foramen, the EPL 25.05 instrument exhibited a diameter of 0.275 mm, causing deviation. It can be inferred that, when the apical patency instrument promotes canal deviation, subsequent root canal preparation tends to follow the same deviated path.

Another factor that could explain the lack of statistically significant apical transportation could be the imprecise synchronization between pre- and postoperative images.^12^ Owing to the image adjustments by made by the software, some edges may have been smoothed, producing artificially regular contours, as also reported by Gambill et al.^4^ Therefore, in addition to selecting a high-quality tomograph for capturing images, it is crucial to use the appropriate software and rely on a well-trained operator.

The present study demonstrated that EPL instruments produced low levels of apical transportation, with no significant differences between the evaluated groups. Wu et al.^5^ pointed out that apical transportation greater than 0.3 mm facilitates the persistence of microorganisms and tissue remnants on dentinal walls, compromising disinfection and the sealing capacity of the root canal system, affecting the quality of the filling. Diaz Zamalloa et al.^24^showed that maintaining apical patency 1 mm beyond the foramen with an EPL 25.01 instrument may result in a greater number of voids in the fit of the master cone, which may negatively influence the filling quality and the prognosis of endodontic treatment.

The clinical relevance of this study has to be constantly reviewed to certify the feasibility of performing apical patency with heat-treated nickel-titanium instruments, mainly those with controlled memory, and their sequential use during mechanical preparation.

## Conclusion

The assessment of root canals in this study using CBCT demonstrated that apical patency and subsequent instrumentation with a CM instrument did not affect apical transportation; however, it may slightly influence the centering ability of the instrument in the root canal.

## Data Availability

The authors declare that all data generated or analyzed during this study are included in this published article.
